# Effects of Intravitreal Ranibizumab Injection on Chinese Patients with Wet Age-Related Macular Degeneration: 5-Year Follow-Up Results

**DOI:** 10.1155/2016/6538192

**Published:** 2016-11-03

**Authors:** Yingyi Lu, Jianfeng Huang, Jing Zhao, Xiaobing Yu, Li Long, Hong Dai

**Affiliations:** Department of Ophthalmology, Beijing Hospital, National Center of Gerontology, No. 1 Dahua Road, Dongdan, Beijing 100730, China

## Abstract

*Purpose.* To observe the effect of intravitreal ranibizumab injection on wet age-related macular degeneration (wAMD) over 5 years in Chinese patients.* Methods.* Thirty-seven patients who were diagnosed with wAMD in our hospital from June 2007 to June 2014 were retrospectively reviewed. The PRN regimen and the treatment and extend regimen were applied. Best corrected visual acuity (BCVA), number of ranibizumab injections, and changes in the choroidal neovascularization (CNV) lesion over 5 years were analyzed.* Results.* The mean BCVA measured by the ETDRS chart at baseline was 47.4 and 5 years after the treatment it was 34.89 letters, which was significantly different (*p* = 0.013). Fourteen eyes (37.8%) had improved visual acuity after 5 years. The number of injections in 5 years was 11.53, and most of the injections were in the first two years. Seventeen (45.9%) cases developed fibrous lesions, and 2 (5.4%) cases had atrophic lesions after 5 years. The fibrosis/atrophy was significantly correlated with the injection numbers (Pearson, *r* = 0.663, and *p* = 0.000).* Conclusion.* Most of the patients can maintain visual acuity treated by ranibizumab in the first 3 years. After 5 years, some patients can still improve or maintain visual acuity. Fibrous scarring of the lesion is the main reason for a decrease in vision of wAMD patients.

## 1. Introduction

Age-related macular degeneration (AMD) is the most important cause of blindness in aged populations in developed countries. As the aging population and average life expectancy increase, the incidence of AMD is growing in developing countries [1]. The efficacy and safety of the anti-VEGF medicine, ranibizumab (Lucentis), to treat wet age-related macular degeneration (wAMD) have been demonstrated in many large samples, multicenter randomized clinical trials, and substantial clinical practice [2, 3]. It is now one of the most widely used treatments for wAMD and choroidal neovascularization (CNV) secondary to many other reasons [4–6]. We are the first to report the use of ranibizumab to treat wAMD in Chinese patients, and the aim of this study is to summarize the long-term effect of ranibizumab on wAMD over a period of 5 years in Chinese patients.

## 2. Materials and Methods

### 2.1. Subjects

Thirty-seven eyes from 37 patients, who were treated with intravitreal ranibizumab injection in Ophthalmology Department of Beijing Hospital from June 2007 to June 2014, were retrospectively analyzed. The inclusion criteria included (1) corresponding to the diagnostic standard of wAMD, (2) being able to be followed up promptly according to the treatment protocol, (3) having a follow-up time of more than 60 months, and (4) no other anti-VEGF drugs or other treatments during follow-up. At baseline, all eyes had an activating lesion, which presented with retinal edema, thickening, or patched retinal bleeding. FFA/ICGA showed CNV leakage. All lesions were smaller than 4 DA, 14 (37.8%) of which were accompanied by PED.

### 2.2. Examination

All patients were examined by the ETDRS chart for visual acuity, ophthalmoscope, FFA, ICGA, and OCT on their first visits. The ETDRS visual acuity, ophthalmoscope, and OCT were examined on every follow-up. FFA/ICGA was selectively examined.

### 2.3. Treatment Method

Intravitreal ranibizumab injection was performed after diagnosis and after informed consent was signed. Ranibizumab (0.5 mg) was injected according to the standard protocol. The anterior segment, IOP, and routine fundus test were examined within the first week after the injection to survey the operation-related complications.

### 2.4. Treatment Protocol and Follow-Up

The treatment was injected monthly 3 times at the loading dose and then every month thereafter. Retreatment was given based on the examination results. In the recent 2 years, the follow-up was instructed by the T&E protocol, in which the follow-up time was gradually extended for 2 weeks up to more than 3 months if the lesion was stabilized. If the lesion relapsed, the follow-up went back to once a month again. In the first two years, according to the standard mentioned in the PrONTO study, we chose to retreat the patients when 2 of the following conditions were met: (1) the BCVA decreased more than 5 letters or the VA decreased subjectively; (2) subretinal or intraretinal fluid accumulation on the macula area was measured by OCT; (3) there was a new hemorrhage lesion on the macula; (4) CNV leakage increased or a new lesion developed according to FFA and/or ICGA. In the late stage, which was 2 years later, we choose to retreat the patients according to the activation of the lesion as follows: relapse or accumulation of intraretinal or subretinal fluid and new onset hemorrhage lesion related to the VA decrease. We performed follow-up in the patients with stable PED or a prolonged intraretinal cyst. The follow-up time was 60–97 months (average 67.4). During follow-up, 5 eyes were injected with a higher dose (1.0 mg, 0.1 mL) for an increasingly shortened relapse interval. The retreatment criteria are patients with persistent recurrent macular intraretinal or subretinal fluid within 30 days.

### 2.5. Statistical Analysis

All data were analyzed by SPSS 16. One-way analysis of variance was used to analyze the BCVA at baseline and at every year after treatment. The relationship between the fibrosis/atrophy of the lesion in the 5th year after treatment and the injection times was analyzed by Pearson correlation analysis. A *p* value < 0.05 was considered statistically significant.

## 3. Results

### 3.1. Patients

**Figure 1:**
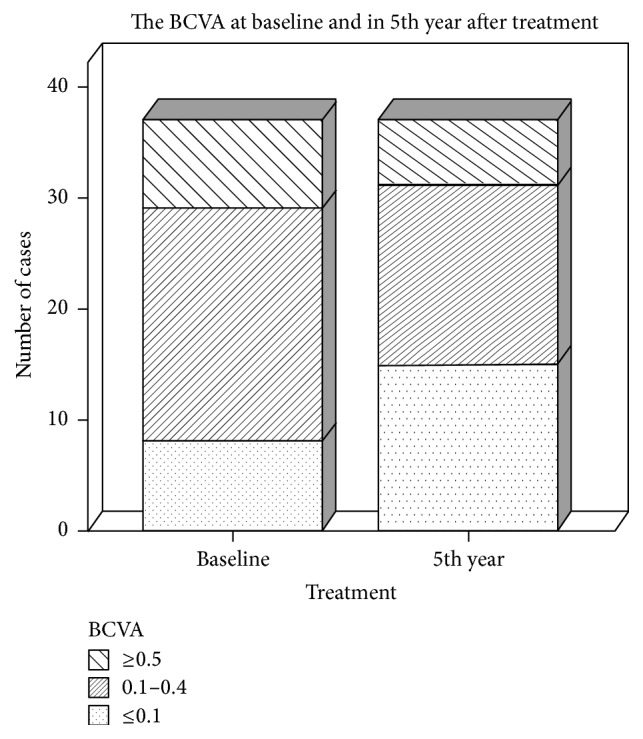
The distribution of the mean BCVAs before treatment and 5 years after treatment.

There were 22 male patients and 15 female patients, and there were 17 right eyes and 20 left eyes. The age of the patients ranged from 59 to 82 years, and the average age was 74.5 ± 6.0 years. Nine of the 37 eyes (24.3%) had a PDT, avastin, or TA treatment history. Twenty-eight eyes were treatment-naïve. The VA of 37 patients before treatment ranged from 0.01 to 0.1. Among them, the VA of 8 eyes (21.6%) was ≥0.5, the VA of 21 eyes (56.8%) was between 0.1 and 0.4, and the VA of 8 eyes (21.6%) was ≤0.1. The mean BCVA examined using the ETDRS chart was 47.4 letters before treatment. Five years after the treatment, the VA of 6 eyes (16.2%) was ≥0.5, the VA of 16 eyes (43.2%) was between 0.1 and 0.4, and the VA of 15 eyes (40.5%) was ≤0.1 (Figure 1). The mean BCVA examined by the ETDRS chart was 34.89 letters (Table 1).

**Figure 2:**
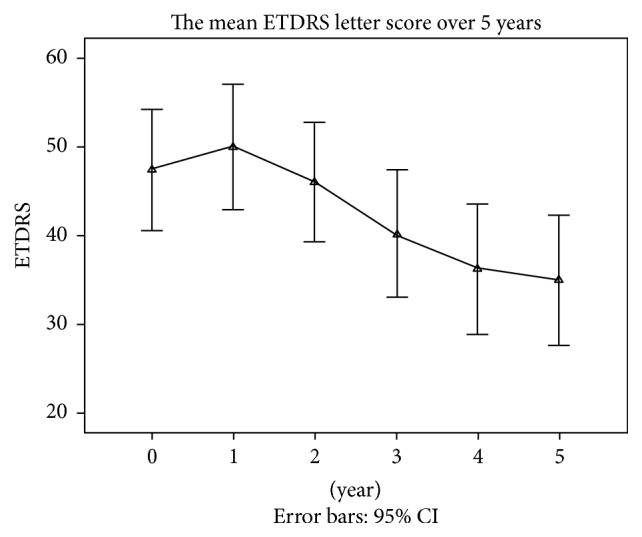
The mean BCVAs examined by the ETDRS chart at baseline and at every year after treatment.

There was no statistically significant difference between the BCVA at baseline and that in the first 3 years after treatment (*p* = 1.314, 0.595, and 0.148). The BCVA at baseline and in the 5th year after the treatment had statistically significant difference (*p* = 0.013). The BCVA in the 5th year after the treatment was significantly different (*p* = 0.024) from that in the first 2 years after the treatment (*p* = 0.03 and 0.025) (Figure 2).

### 3.2. Outcome of the Lesions

The lesions were estimated by fundus image and OCT 5 years after the treatment to check the formation of geographic atrophy and/or fibrotic scar. The lesion was stable at the first year after the treatment in 5 of the 36 patients; the VA increased and there was no recurrence. Twenty-six eyes (70.3%) had no treatment after 3 years because the lesion was stable or became a fibrous scar. Four eyes (10.8%) still needed uninterruptible treatment because of activation of the lesion after 5 years. Five years after the treatment, 17 eyes (47.22%) had fibrosis, and 2 eyes (5.4%) had an atrophic change. The fibrosis/atrophy of the CNV lesion 5 years after the treatment was significantly correlated with the intravitreal injection times (Pearson, *r* = 0.663, and *p* = 0.000).

### 3.3. Treatment Times

The number of injections ranged from 3 to 31 in 5 years, and the average number was 11.53. The injection was concentrated in the first 2 years, and, from the third year on, the injection number was gradually reduced (Table 2). Five patients (13.5%) underwent more than 20 injections, 18 (48.6%) patients underwent 10–19 injections, and 14 (37.8%) patients underwent fewer than 10 injections.

## 4. Discussion

Our previous reports showed that the use of ranibizumab to treat wAMD in Chinese people has clear short-term efficacy and safety [7–9]. The long-term follow-up results of this study demonstrate that although the average BCVA decreased by 13 letters compared with the BCVA at baseline after 5-year treatment with ranibizumab, 37.8% of the patients had improved vision and 13.5% among whom gain 15 letters or more, indicating that the long-term efficacy of ranibizumab to treat wAMD in Chinese people is clear and definite. Our results are similar to the other researches [10, 11]. We found that the average BCVA in the first, second, and third years had no significant difference compared with that at baseline, indicating that most of the patients can maintain their visual function in the first three years, results which are similar to other reports [12]. In the late stage, the average BCVA significantly decreased, which coincided with the change in the CNV lesion. The proportion and extent of fibrosis of the CNV lesion increased with prolonged follow-up. The CNV lesion in 46% of the patients in this study showed fibrosis, while only 5.4% had atrophy. The proportion and extent of fibrosis in Chinese people are much higher than those in other reports [13, 14], whether because some patients had previously undergone PDT treatment or because the different pathophysiological changes in Chinese people are not yet clear. Eyes that received monthly ranibizumab had a higher incidence of geographic atrophy when compared with PRN treatment [15]. This was similar to the results of our PRN treatment, in which the geographic atrophy was less than the fibrosis. This decrease in vision was accompanied by expansion of the size of the total neovascular complex comprising neovascularization, scarring, and atrophy and by persistence of fluid on OCT [15]. We also found that the number of injections is correlated with fibrosis. Fibrosis may be due to multiple factors, and the exact cause has yet to be studied. Fibrous scar is the major reason for a decline of vision in wAMD patients, and reducing or avoiding fibrosis is an effective approach to improving vision, suggesting that combined therapy with antifibrosis might be the new trend for CNV treatment.

Our results demonstrate that the average injection time is nearly 12, and 13.5% of patients were injected more than 12 times, while 37.2% were injected fewer than 10 times. Our average injection time was less than that of CATT study, in which the number was 15.4 [15]. Patients who undergo fewer injections can be classified into 2 subtypes according to the CNV features. First, when the lesion is small, treatment is effective, and patients rarely relapse. Second, when the lesion becomes a fibrous scar, the treatment is stopped. From a statistical point of view, most injections occur during the first year and second year, which is related to the increase in CNV fibrosis in some of the patients. Our results show that 10% of patients need treatment after 5 years due to lesion activation, indicating that some wAMD patients require long-term, continuous treatment [9]. In some patients, the repetitive injection interval is shorter with increases in the injection times, and we increased ranibizumab dose to 1.0 mg, which can extend the efficacy period, suggesting that some patients may be resistant to anti-VEGF medicine after long-term application. Another study from our group indicates that the probable anti-VEGF antibody can affect the efficacy [16–18]. During treatment, an individualized regime that is based on the patient's reaction is needed [19, 20]. For anti-VEGF drugs that have poor outcome, increasing the dosage, changing the drug, and combined treatment can be applied [21].

In the WAMD treatment guidelines, the follow-up time for patients is recommended as once per month. In clinical practice, especially during long-term follow-up, once per month is a high burden for both patients and doctors [22]. In the early stage, the patients are followed up once a month, for those whose CNV lesion is relatively steady and who can use the Amsler grid by themselves, so the T&E follow-up program is adopted. In the cases where there are stable visual acuity, an absence of macular hemorrhage, and a dry OCT, the follow-up intervals increase to 6 weeks without treatment. If there are no changes, the next visit is scheduled for 8 weeks later. If there is a change, the patient comes for injection and examination after 4 weeks. Practice shows that this program not only reduces the number of follow-up visits but also can promptly detect the change in the CNV, suggesting that it can be used in clinical practice.

wAMD patients commonly have age-related cataracts [23]. With the increase in the number of injections or prolonged follow-up time, cataracts will develop and impair the patients' visual function. Patients and doctors should be concerned about whether cataract surgery can improve the vision of wAMD patients, whether the surgery can influence the CNV lesion, and when to perform the surgery. In our study, 7 cases underwent cataract surgery combined with IOL implantation during the 5 years of follow-up, and most of the patients had improved visual function or visual clarity. Another study of ours demonstrated that half of the wAMD patients have improved visual acuity after cataract surgery, and 70% of the patients have improved visual clarity or visual field vision. The immediate postoperative OCT examination did not reveal any CNV lesion changes. For some patients, the anti-VEGF drug injection and cataract surgery were performed simultaneously, and there was no interaction between them. In wAMD patients, if a significant cataract develops, surgery can be performed even if the CNV lesion is active. However, because of the uncertainty of improved visual acuity after cataract surgery, a preoperative visual prognosis and sufficient explanation are needed.

Although the impact on visual acuity is multifactorial during long-term follow-up and the implementation of the treatment program is not uniform because different doctors and patients can affect the results of this study, our data reflects clinical treatment and can provide a reference for clinical use.

## Figures and Tables

**Table 1:** Visual acuity gain or loss at the 5th year after treatment.

Visual acuity ETDRS	VA at the 5th year after treatment (*n* = 37), *n*%
Gain ≥ 15 letters	5 (13.5%)
Gain within 15 letters	9 (24.3%)
Loss within 15 letters	10 (27.0%)
Loss ≥ 15 letters	13 (35.1%)

**Table 2:** The mean treatment times in 5 years.

Follow-up visit	Total number receiving injection (*n* = 37)
1st year	5.93 ± 4.20
2nd year	3.41 ± 0.78
3rd year	0.91 ± 1.11
4th year	0.74 ± 1.40
5th year	0.54 ± 1.25
